# Social Media Interventions for Risky Drinking Among Adolescents and Emerging Adults: Protocol for a Randomized Controlled Trial

**DOI:** 10.2196/16688

**Published:** 2020-05-13

**Authors:** Erin E Bonar, Diane M Schneeberger, Carrie Bourque, Jose A Bauermeister, Sean D Young, Frederic C Blow, Rebecca M Cunningham, Amy SB Bohnert, Marc A Zimmerman, Maureen A Walton

**Affiliations:** 1 Department of Psychiatry University of Michigan Ann Arbor, MI United States; 2 Addiction Center University of Michigan Ann Arbor, MI United States; 3 Injury Prevention Center University of Michigan Ann Arbor, MI United States; 4 Department of Family and Community Health School of Nursing University of Pennsylvania Philadelphia, PA United States; 5 Department of Informatics Donald Bren School of Information and Computer Sciences University of California Irvine Irvine, CA United States; 6 Department of Emergency Medicine University of California Irvine Irvine, CA United States; 7 Center for Clinical Management Research Veterans Health Administration Ann Arbor, MI United States; 8 Department of Emergency Medicine University of Michigan Ann Arbor, MI United States; 9 Health Behavior & Health Education School of Public Health University of Michigan Ann Arbor, MI United States

**Keywords:** social media, alcohol consumption, adolescents, emerging adults, internet-based intervention

## Abstract

**Background:**

Despite intervention efforts to date, the prevalence of risky drinking among adolescents and emerging adults remains high, increasing the risk for health consequences and the development of alcohol use disorders. Peer influences are particularly salient among this age group, including via social media. Thus, the development of efficacious early interventions for youth, delivered with a broad reach via trained peers on social media, could have an important role in addressing risky drinking and concomitant drug use.

**Objective:**

This paper describes the protocol of a randomized controlled trial (RCT) testing the efficacy of a social media intervention among adolescents and emerging adults who meet the criteria for risky drinking (using the Alcohol Use Disorders Identification Test-Consumption [AUDIT-C]), delivered with and without financial incentives for participation, compared with an attention placebo control condition (ie, entertaining social media content), on alcohol consumption and consequences.

**Methods:**

This RCT involved recruiting 955 youths (aged 16-24 years) via advertisements on Facebook and Instagram to self-administer a brief web-based screening survey. Those screening positive for past 3-month risky drinking (AUDIT-C positive: ages 16-17 years: ≥3 females and ≥4 males; and ages 18-24 years: ≥4 females and ≥5 males) were eligible for the RCT. After providing consent (a waiver of parental consent was obtained for minors), participants completed a web-based baseline survey and several verification procedures, including a selfie photo matched to Facebook profile photos. Participants were then randomized to join invitation-only secret Facebook groups, which were not searchable or viewable by parents, friends, or anyone not recruited by the study. The 3 conditions were social media intervention with incentives, social media intervention without incentives (SMI), and attention placebo control. Each condition lasted 8 weeks and consisted of bachelor’s-level and master’s-level therapist electronic coaches posting relevant content and responding to participants’ posts in a manner consistent with Motivational Interviewing. Participants in the control condition and SMI condition did not receive payments but were blind to condition assignment between these 2 conditions. Follow-ups are ongoing and occur at 3, 6, and 12 months poststart of the groups.

**Results:**

We enrolled 955 participants over 10 waves of recruitment who screened positive for risky drinking into the RCT.

**Conclusions:**

The findings of this study will provide the critical next step in delivering early alcohol interventions to the youth, capitalizing on social media platforms, which could have significant public health impact by altering alcohol use trajectories of adolescents and emerging adults engaged in risky drinking.

**Trial Registration:**

ClinicalTrials.gov NCT02809586; https://clinicaltrials.gov/ct2/show/NCT02809586.

**International Registered Report Identifier (IRRID):**

DERR1-10.2196/16688

## Introduction

### Background

Despite numerous intervention and policy efforts, risky drinking (ie, hazardous levels of consumption resulting in increased risk for consequences) among youth in the United States remains a major public health issue. Although only 1.8% of youths aged 12 to 17 years and 10.0% of those aged 18 to 25 years met criteria for an alcohol use disorder in 2017 in the United States [1], risky drinking is common. For example, as one indicator of risky drinking, past-month binge (eg, ≥4 drinks for females and ≥5 drinks for males) drinking rates are 10.2% for ages 16 to 17 years, 26.2% for ages 18 to 20 years, and 45.4% for ages 21 to 25 years [2], although these may be underestimates because of possible underreporting in national surveys (eg, average past-year quantity or frequency questions) [3]. In fact, risky drinking among young people is associated with increased risk for other drug use, adverse health consequences (eg, injury and overdose), and development of substance use disorders [4-7]. Accordingly, late adolescence and emerging adulthood is a critical developmental juncture, distinct from childhood and adulthood, during which rates of alcohol and other drug use peak [8-10]. For example, 6.5% of adolescents and 22.1% of emerging adults report past-month cannabis use [1]. Thus, scalable, early interventions are urgently needed to address risky drinking and concomitant health risk behaviors (eg, other drug use and driving under the influence) among adolescents and emerging adults to disrupt risk trajectories. Here, we present the theoretical rationale and protocol for a randomized controlled trial (RCT) of social media–delivered interventions for risky drinking among adolescents aged 16 to 24 years recruited nationally.

### Conceptual Model

The conceptual model guiding our intervention is rooted in social cognitive (eg, theory of planned behavior [11] and social learning [12]) and social ecological [13] theories, emphasizing the role of individual and social influences on alcohol use by adolescents and emerging adults. Furthermore, our intervention is implicitly grounded in a resiliency framework [14,15]. Across development, evolving interactions between individual and social risk and protective factors during the establishment of new roles (eg, relationships and employment) [16] can decrease or accelerate alcohol use trajectories [16-18]. Individual risk factors associated with alcohol use include low perceived risk of use, perceived norms, and mental health issues (ie, depression and anxiety), whereas disapproval of use, parenting practices, and protective behavioral strategies are protective [16,19-27]. Although parents are important during younger ages [28], peers comprise the most robust social influences on substance use among adolescents and emerging adults [16,29-38].

Over the past decade, social media has become increasingly prevalent in the day-to-day lives of young people, creating additional opportunities for exposure to positive and negative peer influences [39,40]. Social media content is user generated and constantly changing, providing frequent exposure to web-based peer influences, potentially resulting in reinforcing spirals of increasing exposure and involvement with alcohol use behaviors over time [41,42]. Although it is well known that offline peers can exert tremendous influence on alcohol use among youth [16,29-32], recent data suggest that online peers also influence alcohol use [43-45]. Emerging adults who consume more alcohol, for example, have more Facebook friends [46] and post more references to parties on Facebook than those who use less alcohol [47]. Among high school students, higher alcohol use is related to reports of friends posting alcohol content on social media [43], and in a laboratory study, researchers found that teens viewing Facebook profiles that contained positive references to alcohol had more positive attitudes and willingness to drink alcohol than teens who did not view these profiles [48]. As offline peer disapproval of risky substance use can be a protective factor [49,50], online peer disapproval of alcohol use may function similarly. Research shows that posting positive portrayals of alcohol use on social media is related to consumption among the youth [43,51]. Thus, social media provides an appealing platform for the delivery of alcohol interventions, wherein peer influences could be harnessed instead to promote harm reduction or reduced consumption. As described earlier, alcohol use is associated with other drug use; thus, social media interventions could be useful for targeting concomitant drug use, particularly because mentions of other drug use are also prevalent on social media [52,53]. For example, one study showed that more than one-third of a college student sample had seen a picture of a friend smoking cannabis posted on social media [54].

### Social Media as an Intervention Platform

A common feature of social media use among adolescents and emerging adults is the frequent use of more than one platform, which reflects increased smartphone ownership and Wi-Fi access. As of 2018, 95% of teens reported having a smartphone or access to one, of which 45% reported they are on the Web almost constantly [55]. Among emerging adults (aged 18-25 years), 88% use Facebook, compared with 68% for Snapchat, 59% for Instagram, and 36% for Twitter [56]. In addition, engagement is more frequent for Facebook, with 74% of all users checking it daily, compared with 61% for Snapchat, 63% for Instagram, and 42% for Twitter [56], and 51% of Facebook users log in several times per day [57]. In contrast, among adolescents (aged 13-17 years), 51% use Facebook (which has declined in recent years) [58], compared with 69% for Snapchat, 72% for Instagram, and 32% for Twitter [56]. It may be that adolescents and emerging adults who use Facebook regularly differ from those who do not, which could affect the utility of interventions. For example, data suggest that a larger proportion of teens from lower income households use Facebook than those from higher income households [59]. Thus, when choosing a social media intervention platform to reach both adolescents and emerging adults, there is no clear single best choice. Furthermore, as trends in social media use shift over time and/or within demographic groups, there may be unique opportunities to leverage content for delivery across various emerging platforms with shared features (eg, ability to post personal content, videos, articles, etc) to reach certain at-risk groups.

To date, there a very few social media interventions to reduce risky drinking (and/or other illicit or prescription drug misuse) among young people [60], with a recent publication describing the development of a tobacco and binge drinking intervention [61]. Most prior research testing early interventions for alcohol (and other drug use) has examined interventions delivered by therapists and/or static computer programs, with demonstrated efficacy in medical and university settings [62-74]. Overall, effect sizes are modest [75], with newer studies in the substance use field and other health fields testing technology-driven methods to extend delivery [76-79]. An advantage of social media interventions is that they can be designed to blend therapist and computerized interventions to deliver dynamic, evolving content; harness online peer influences; and provide access to electronic coaches (e-coaches), which can increase exposure to content at the time the person chooses to engage. Social media interventions (typically delivered over 8-12 weeks), addressing other health outcomes (eg, exercise/weight, HIV risk reduction/sexual health, and smoking cessation) among varied samples (eg, postpartum women, college students, and general community), have demonstrated promising effects [80-88], supporting the potential of this approach to address alcohol and other drug use.

Prior social media interventions have used Facebook for delivery, likely because it remains the most popular social media site among emerging adults and it has unique features that support intervention delivery. For example, Facebook allows private, secret groups to address privacy and confidentiality concerns (which are not searchable or viewable by others and can be joined by invitation only). In addition, the content is sorted into threads, promoting group interaction, with active conversations bumped to the top of the group or one’s newsfeed. Moreover, Facebook content does not disappear (eg, as in Snapchat), so it can be viewed an unlimited number of times and discussions can be revisited, as group members post new comments. Finally, Facebook does not restrict the character count of posts, which is a limitation of other platforms.

Critical issues related to designing social media interventions are exposure, dose (engagement or response showing the degree to which content may be processed), and diffusion (reach or interaction among online peers via shares, comments, etc) [89]. Exposure can be measured using metrics of reactions (eg, likes), comments, replies, and posts to Facebook groups. For instance, researchers found more than half (approximately 63%) of participants in a physical activity intervention condition reported visiting the Facebook group 2 to 3 times per month during a 12-week intervention period; among those who posted in the Facebook group more than once, they averaged 8 interactions each over 12 weeks [80]. Thus, an important methodological question is related to how to encourage engagement, increasing dose and diffusion [89]. Our study sought to accomplish this in 2 ways. First, content was informed by social marketing research tips regarding characteristics of posts that increase interaction: (1) give (eg, photo/video contests), (2) advise (useful tip for concerns, eg, coping strategies), (3) warn (dangers could affect anyone, eg, overdose and impaired driving), (4) amuse (amusing photos/videos), (5) inspire (moving quotes or stories), (6) amaze (amazing pictures or facts, eg, norms), and (7) unite (brag about group membership and social support) [90]. Second, to our knowledge, no researchers have compared intervention conditions that vary incentives for engagement. Increased interaction via incentives among peers could theoretically reinforce group interactions, increasing dose, which is thought to result in behavior change. Thus, we sought to compare an intervention condition that provided modest financial incentives for engagement as measured by daily interactions (ie, posts with status updates or comments to another’s post) with a condition that did not provide incentives. Thus, in addition to comparing the interventions to an attention control condition to determine efficacy, our goal was to examine whether externally incentivized interaction produced greater engagement, and if so, whether that enhances intervention efficacy, relative to the nonincentivized intervention condition containing organic, individually motivated interactions.

Finally, sentiment analysis (eg, examining the relative positive or negative valence [tone] and arousal [activation] in text) [91,92] is a potentially useful tool to understand characteristics of engagement in social media interventions [93]. Using state-of-the-art software, natural language processing can evaluate slang and common misspellings, with 85% accuracy [94,95]. To date, sentiment analysis has been applied to smoking cessation interventions but has not been applied to alcohol interventions, although researchers are coding content of social media related to alcohol use [96,97]. Thus, because our interventions sought to encourage interaction within secret groups, sentiment analysis is an innovative approach to understanding the characteristics of engagement (eg, valence and arousal) and alcohol use outcomes.

**Goal of This Study**

We recruited adolescents and emerging adults using Facebook and Instagram advertisements and conducted web-based screening to enroll risky drinkers in an RCT comparing 3 conditions: (1) social media intervention (SMI) with incentives (SMI+I), (2) SMI only, and (3) attention control condition, with follow-up assessments at 3, 6, and 12 months. Interventions comprised access for 8 weeks to unique, private secret Facebook groups facilitated by e-coaches (supervised by licensed therapists), with dynamic content addressing motives for risky drinking and reducing consumption as well as concomitant risk behaviors (other drug use). The attention control condition included access for 8 weeks to entertaining content (eg, sports, lifestyle, fun, etc). As described earlier, we used 2 intervention approaches, with and without financial incentives for participation, and we will measure engagement within the intervention groups. By providing incentives for participation in one condition, we attempted to harness participants to provide group support, thereby delivering intervention content, facilitated by e-coaches.

The specific aims are to (1) test the efficacy of the 2 intervention conditions compared with the control, in reducing alcohol consumption and alcohol-related consequences at 3-, 6-, and 12-month follow-ups; (2) compare the intervention conditions in participant engagement and efficacy in reducing alcohol consumption and alcohol-related consequences at 3-, 6-, and 12-month follow-ups; and (3) examine how level of engagement in intervention conditions (eg, engagement metrics) and characteristics of intervention engagement (sentiment analysis) relate to alcohol use outcomes in the 2 intervention conditions. The secondary aims include examining the efficacy of the interventions on other drug use, moderators of outcome, and conducting cost analyses. This paper describes the study protocol in relation to the primary aims.

## Methods

### Trial Registration, Ethics, Consent, and Institutional Board Approval

The study procedures were approved by the University of Michigan Institutional Review Board (IRB), and the study was registered in Clinicaltrials.gov (#AA024175). We received a waiver of parental consent for all aspects of the study for youths aged 16 to 20 years (the age of majority varied based on state residence). The rationale for this waiver was based on (1) the determination of teenage participants as *mature minors* (ie, they can understand the study risks), with decisional capacity to promote health-seeking behavior including substance use treatment [98]; (2) the fact that disclosure of high-risk behaviors may increase the risk of adverse effects on participants’ well-being because of potential reactions from parents (eg, rejection and abuse); and (3) the study could not practicably be carried out without this waiver, given potential bias in participation because of fear of disclosure of risky drinking to parents [99]. Furthermore, our study involved a two-phase consent process, with separate web-based consent obtained for the screening and RCT phases. Confidentiality and privacy were also enhanced by requiring participants to agree to abide by our own User Safety Agreement (see the *Interventions* section). We obtained a Certificate of Confidentiality from the National Institutes of Health.

### Design

Using recruitment via social media advertisements, we enrolled and randomized 955 adolescents and emerging adults (aged 16-24 years) in an RCT comprising the 2 intervention conditions and the control condition. Participants were assigned their conditions for a period of 8 weeks and were prompted to self-administer follow-up assessments at 3, 6, and 12 months postinitiation of groups. All assessments and interventions occurred on the Web, with surveys administered through Qualtrics [100].

### Recruitment

Potential participants were recruited in 10 waves, separated by age (16-20 and 21-24 years) via paid advertisements on Facebook and Instagram. Each wave contained an average of 95.5 participants, which helped ensure that the 3 groups in each wave contained approximately 30 participants (mean=31.8 participants per group) to allow for sufficient online group interaction.

On the basis of prior work [101], Facebook/Instagram advertisements were initially placed by setting the audience location to include users in the United States. Advertisements were also specified to be displayed to users with certain demographic characteristics (ie, age groups 16-17, 18-20, 21-22, and 23-24 years and English-speaking users) and detailed targeting displayed advertisements to users who *liked* Facebook pages related to alcohol (eg, popular brands, drinking games, etc). Starting in wave 5, we added user characteristics to increase the recruitment of racial and ethnic minorities using affinity targeting within Facebook Ads Manager. After wave 6, ethnic affinity targeting was temporarily removed from the Facebook Ads Manager but was available again and used for waves 9 and 10. Each advertisement featured headlines to encourage potential participants to take the survey (eg, “Drink alcohol? Participate in a research study; earn $$$ for your time.”). We used 3 images (from Facebook Ads Manager and stock photos) of alcohol/individuals with alcohol and one image of the study logo. To encourage minority representation in the sample, advertisements pictured individuals of varying races/ethnicities. Also starting in wave 5, white individuals and females were informally excluded after preset quotas were filled. Advertisements initially directed participants to the study website, but starting in wave 2, advertisements led participants directly to the consent page and screening survey. The study’s website URL was provided to participants throughout the study (eg, in Facebook secret groups and texting/email communications).

### Screening

Among screening-eligible participants, the 21-item web-based screening survey was used to determine RCT eligibility using a past 3-month version of the Alcohol Use Disorders Identification Test-Consumption (AUDIT-C [102-104]; where binge drinking was defined at ≥4 drinks for women and ≥5 drinks for men) embedded among other standard items querying demographics and other substance use. To ensure real people completed the survey (as opposed to bots), the web-based screening consent page included a Completely Automated Public Turing test to tell Computers and Humans Apart. Participants who reported risky drinking the past 3 months (AUDIT-C score: ages 16-17 years: ≥3 females and ≥4 males and ages 18-24 years: ≥4 females and ≥5 males [105,106]) were eligible for the study.

### Participant Identity Verification Procedures and Baseline Enrollment

Before enrolling eligible participants, we reviewed their screening data as a second step to ensure data integrity and to deter fraudulent participation. Procedures included checking data for duplicate internet protocol addresses, survey completion times >60 seconds, and the existence and legitimacy of the participant’s Facebook profile based on published recommendations [107,108]. Once initial identity verification procedures were passed, eligible youths were sent an email invitation to participate in the study, with a link that automatically directed them to the RCT consent form followed by a web-based baseline survey and a contact information form. Participants were informed in the consent form that “the purpose of the study is to develop and test social media interventions to help young people reduce risky behaviors, such as alcohol use.” To help ensure identity and age, as part of the baseline procedures, participants were required to upload a selfie containing a handwritten sign with the date and time that included their head and shoulders. Study staff compared the selfie with the participant’s Facebook profile for verification before randomization and group assignment. In rare cases where a participant’s Facebook profile did not already contain a photo of themselves, we asked them to temporarily upload a second photo (different than the selfie) for real-time, immediate verification against their time- and date-stamped photo.

### Randomization

Following the web-based baseline assessment and selfie verification, participants were randomized to 1 of the 3 conditions. Given differences in severity of drinking by age and sex, which could affect response to the intervention [2], computerized, stratified random assignment by sex and age group (16-20 and 21-24 years) took place within condition, in blocks of 20 within cells to equalize randomization over time. Randomization occurred by a computer algorithm generated with supervision by the data manager; thus, research staff were not able to manipulate condition assignment. Given e-coach interaction in groups, it was not possible to blind staff to condition assignment; regarding participant blinding, although participants were not told whether they were assigned to an intervention or control group, the control group did not receive alcohol content; thus, as with most behavioral trials, it is possible that participants discerned their condition assignment. Specifically, the consent form described the 2 intervention conditions, “You will have access to the secret Facebook page that will deliver health information focused on reducing risky behaviors, including alcohol use.” The consent form described the control condition as, “You will have access to the secret Facebook page that will share news information about things like entertainment, sports, weather and world news.” In addition, for the SMI+I group, participants were informed that they would earn points for interacting on the group page and be paid for the points earned, so participants were not blinded to being assigned to the payment condition. After randomization, participants were sent a friend request from an e-coach; once participants accepted the request, they were added to their corresponding secret group where the 8-week condition was delivered.

### Follow-Up Assessments

Consecutive web-based assessments, mirroring the baseline survey measures, were distributed by a research assistant using a generic study email address at 3, 6, and 12 months after group initiation. Participants were assured that e-coaches would not view their individual outcomes on their self-administered follow-up surveys.

### Incentives

Each participant received a US $30 Amazon gift card code for completing the web-based baseline survey and providing a selfie, which took approximately 30 min. Compensation for follow-up assessments was US $35 for the 3-month assessment, US $45 for the 6-month assessment, and US $55 for the 12-month assessment. Participants in the SMI+I condition received incentives to encourage interaction, earning US $1.00 for each day they posted text and/or images in the secret group (ie, status update, comment, reply, or share) for a maximum of US $56 per participant over 8 weeks. Note that *likes* or *reactions* (eg, heart and sad face) were not incentivized. Incentives were paid weekly via an electronic Amazon gift card by study staff (student research assistants, e-coaches, and/or supervisors) who reviewed posting data to confirm the number of days on which participants posted.

### Interventions

#### Overview

The interventions consisted of interactions among participants and e-coaches within the secret Facebook group pages (separated by age group: 16-20 and 21-24 years) over 8 weeks, among approximately 30 participants per group. After 8 weeks, participation in the group ended; the ability to share new posts was turned off, but participants could still view archived content. At RCT consent, participants were required to agree to our User Safety Agreement, which provided rules of engagement for the group. These rules included prohibition of posting opportunities to engage in alcohol or other drug use (eg, parties and selling drugs) or obscene or offensive material, advertisements for making money or a business, maintaining participants’ confidentiality, and treating each other with respect. Participants were informed that a single violation of the agreement would result in a reminder of these rules and that repeated violations could result in removal from the group page with redirection to an individual page so that content would still be viewable. Participants were allowed to friend each other and send messages to one another at their own discretion; we did not provide specific User Safety Agreement guidelines for these private interactions.

#### Electronic Coach Training and Supervision

E-coaches were trained in Motivational Interviewing (MI) and Cognitive Behavioral Therapy (CBT) skills and posted and responded to participants in a manner consistent with MI, supervised by licensed clinical supervisors in weekly individual and group supervision [109]. E-coach training included participation in a large group (not study specific), 2-day interactive introductory training in MI led by the first author and other members of the MI Network of Trainers (MINT), one day of small group study-specific MI training with a MINT trainer, one day of small group study-specific MI training with the study coordinator, and completion of 4 web-based MI modules. Supervision included review of groups and collaborative responding to participants’ comments, replies, statuses, or shares. During each 8-week intervention period, group supervision lasted 1 to 2 hours per week, and individual supervision lasted 1 to 2 hours (based on e-coach experience and/or amount of interaction occurring in the groups at a given time). In addition, depending on e-coach skills and the volume and clinical complexity of each wave, a supervisor would post with the e-coach for 1 to 2 hours weekly.

#### Intervention Model

Strategies from CBT [110] were structured in 3 phases: *Explore, Guide, and Choose* [111,112]. Self-determination theory (SDT) [113] is conceptualized to explain how MI works [114]; as applied to alcohol interventions, SDT would suggest that to increase intrinsic motivation to reduce alcohol use, the provider must assist the participant in increasing confidence, relatedness, and autonomy. Within each weekly topic, as part of *Explore*, e-coaches explored risk perceptions, concerns, motives, and current alcohol use along with personal goals and strengths. As part of *Guide*, e-coaches used an Elicit-Provide-Elicit framework, posting open-ended questions and responding to posts by participants, with the goal of eliciting change talk to reduce risky drinking. As part of *Choose*, CBT skills and protective behavioral strategies were elicited (eg, anticipating the consequences of use, finding alternative strategies to address motives for use) and reinforced. When the need arose, e-coaches provided community resources within the group and in private messages. A list of national resources was also available along with a copy of the consent form and User Safety Agreement within the group in a downloadable files section. Finally, a crisis text line and a reminder to call 911 for immediate emergencies were shown in the group cover photo pinned to the top of the secret Facebook page, along with a message that groups were not monitored 24/7 (although they were monitored multiples times per day).

Initially, we developed a prototype of the 8-week intervention based on theory, prior work [115], and feedback from youth advisors, who reviewed initial content in a focus group. To increase our library of content, we used Amazon’s Mechanical Turk to crowdsource content appealing to the youth. Then, we refined the content and focus tested it with another group of youth advisors, who participated in a mock intervention group, followed by content editing. Although the content topics were consistent across waves (see Table 1), the intervention was flexible to address current events (eg, overdose death of a celebrity) and topics initiated by group members, which included topics such as personal struggles or celebrations. Consistent with expectations on social media, posts included links to engaging content (eg, memes, GIF images, Buzzfeed articles, YouTube videos, quizzes/polls, and other web-based articles) paired with evocative statements and questions to encourage participants to interact.

**Table 1:** Weekly content topics addressed in the social media interventions.

Week	Topic	Goal of weekly topic
1	Dealing with stress	Establish rapport, enhance coping to manage stress, affirm personal strengths, and elicit long-term goals
2	What young people do	Explore peer norms, elicit benefits of avoiding/reducing drinking, and enhance self-efficacy for harm reduction
3	Staying out of trouble	Elicit negative consequences of alcohol use and protective behavioral strategies
4	Handling tricky situations	Elicit motives for drinking and reinforce strategies to address motives in healthier ways
5	Free time activities	Elicit free time activities that promote healthy and valued activities while avoiding/reducing drinking
6	Friends and parents	Elicit strategies for managing relationships and situations with others
7	Staying healthy	Elicit skills to prevent life-threatening outcomes of drinking (overdose and drinking/drugged driving)
8	Getting support	Engage participants in identifying resources and promote healthy social support

Multiple times per day for 56 days, e-coaches posted new, dynamic content during morning, afternoon, and evening shifts. The same content was posted by e-coaches across both SMI and SMI+I conditions, at the same daily intervals, with some content tailored by age group. For example, posts in the younger group tended to reference school and parents, whereas posts in older groups tended to mention employment and partner relationships. During daily shifts, e-coaches used MI to respond to participants’ posts and comments. In addition, e-coaches used Facebook tagging and/or sent messages to participants via text, email, or private message if they did not engage for 7 days, sharing trending topics being discussed on the intervention page with the goal of increasing participation. After initial icebreaker posts (eg, How would you describe yourself in 5 words?), the intervention primarily addressed upstream motives for alcohol use (eg, stress, negative affect, positive affect, social influences). Given our secondary aims and in recognition of the harmful health effects of combined alcohol and other drug use, other drug use was also addressed (eg, risk of overdose, drugged driving). As cannabis (followed by misuse of prescription drugs) is the most commonly reported illicit substance used by adolescents and emerging adults [116], we also addressed these other substances throughout the intervention, given the likelihood that many participants could be co-using and experience heightened risks (eg, greening out, overdose). Finally, e-coaches posted weekly polls to assess participants’ content preferences while also monitoring the ongoing popularity of posts for tailoring in future weeks and waves.

### Attention Placebo Control Condition

Similar to prior work [86], participants in the control group were given access to an 8-week attention placebo entertainment condition using private secret Facebook groups. Weekly topics included posts related to nonalcohol or drug-related topics that involved entertaining content (eg, sports, lifestyle, fun, etc). E-coaches posted content within the groups daily, at the same intervals as the intervention group posts. As in the intervention groups, the User Safety Agreement was enforced, the crisis line information was displayed in the group cover photo 24/7, and the downloadable files section included national resources (eg, suicide hotlines, mental health, and substance use treatment), the User Safety Agreement, and a copy of the consent form.

### Outcomes

#### Alcohol Use

Our primary outcome of changes in alcohol consumption (eg, quantity, frequency, binge drinking) in the past 30 days is based on a self-administered, web-based, Timeline Follow-Back (TLFB) assessment [117-119]. We programmed this self-administered measure to embed within our Qualtrics baseline and follow-up surveys, with data housed on our secure internal servers.

#### Alcohol Consequences

Alcohol consequences were measured via the Brief Young Adult Alcohol Consequences Questionnaire (BYAACQ) [120], which asked participants about experiences with 24 specific alcohol-related problems (eg, blackouts, hangovers) over the last 3 months (responses: *0=none* to *3=more than 5 times*). Note that we modified the BYAACQ by removing 2 items that are not frequently endorsed (ie, “My physical appearance has been harmed by my drinking” and “I have felt like I needed a drink after I’d gotten up [that is, before breakfast]”); we substituted 2 additional questions adapted from the original Young Adult Alcohol Consequences Questionnaire (ie, “I have damaged or lost property after drinking” and “I have gotten into physical fights because of drinking”) [121,122].

#### Condition Engagement and Sentiment Analysis

Measures of intervention engagement include counts of engagement data (eg, posts, status, comments to others’ posts, likes/shares). We expect that engagement level will mediate drinking outcomes, in that those who were more engaged in the intervention may respond more positively to the intervention. To examine characteristics of engagement, we will conduct sentiment analysis using software to code valance and arousal (eg, Dictionary of Affect in Language, Affective Norms for English Words) [94,95]. When initially starting the study, we considered using third-party applications to collect these data; however, our IRB did not allow for storing participant identities on third-party servers. Thus, we developed our own automated software application housed on our internal servers to count each user’s likes/reactions, status updates, shares, replies, and comments to monitor engagement (and to assist in calculating incentive payments in the SMI+I condition) and to code sentiment within secret groups. However, midway through the study, Facebook restricted access to our program and all other third-party applications to secret groups. Therefore, study staff hand coded engagement to provide weekly incentives to participants in the SMI+I condition. To complete sentiment analyses (eg, code valence and arousal) and calculate engagement totals, we are currently revising our automated software application to code group conversations.

#### Intervention Acceptability and Perceived Helpfulness

At the 3-month follow-up, as done in prior work [123], participants were asked to rate perceived helpfulness of interactions with Facebook groups and e-coaches and the 8 weekly intervention topics. Example items include, “How helpful was it to interact with other peers in the group?” and “I felt the e-coaches understood me,” with response options ranging from *not at all* to *extremely*. The SMI+I condition received additional questions to assess the perception of incentives for engagement. Finally, the 12-month survey asked participants how many friends they made from the group that they still keep in touch with, with response options ranging from none to 21 or more.

#### Secondary Outcomes of Other Drug Use

Several measures were collected at baseline and follow-ups, which may be used to explore the impact of the interventions on other drug use as a secondary outcome. First, the TLFB described earlier also assessed past 30-day daily cannabis use. In addition, we made minor modifications to items from the Tobacco, Alcohol, Prescription medications, and other Substance tool to query other substances used in the past 3 months [124].

### Statistical Analyses for Primary Aims

We will use generalized linear mixed models (GLMMs) to examine treatment effects and changes in the dependent measures (for both primary and secondary outcomes). We chose GLMMs for 2 primary reasons: (1) GLMMs adjust for correlations between data points (eg, repeated measurements on individuals); and (2) within GLMMs, one can retain participants who do not complete all follow-up assessments in analyses. As the primary outcome variable, alcohol consumption, is unlikely to have normally distributed errors and is effectively integer valued, the Poisson distribution, allowing for overdispersion [125], is a natural choice. This assumption will be scrutinized, and, as needed, modifications (eg, zero inflation) [126] and alternative families of distributions (eg, negative binomial) will be considered. For models treating level of interaction, quantified by engagement metrics, as the dependent variable (aim 2), we do not have any *a priori* judgments about the appropriate distributional family, and this will be assessed based on the observed distribution. Our initial choice will be the Gaussian (normal) distribution. In all cases, we will implement an intent-to-treat analysis [127]. Analyses pertaining to secondary outcomes of other drug use will be conducted in parallel manner to the primary outcomes analysis.

#### Aim 1: Develop and Test the Efficacy of Intervention Conditions (Social Media Intervention With Incentives and Social Media Intervention Only) Compared With Control, in Reducing Alcohol Consumption and Alcohol-Related Consequences

##### Hypothesis

Compared with the control group, the intervention conditions will have significantly less alcohol use and consequences.

##### Statistical Analysis

We will assess intervention effects at 3-, 6-, and 12-month follow-ups using the bivariate analyses comparing the 2 intervention conditions to the control condition. We will then examine treatment effects using a Poisson GLMM to account for correlations between repeated measurements. If preliminary bivariate analyses suggest that the effect of the intervention may vary over time, we will model an interaction of intervention by time.

#### Aim 2: Compare the Intervention Conditions (Social Media Intervention With Incentives and Social Media Intervention Only) in Participant Engagement and Efficacy in Reducing Alcohol Consumption and Alcohol-Related Consequences at Follow-Up

##### Hypotheses

Compared with participants in the SMI condition, participants in the SMI+I condition will have (1) greater levels of involvement and (2) have significantly less alcohol use and consequences.

##### Statistical Analysis

As mentioned earlier, we will assess intervention effects at 3-, 6- and 12-month follow-ups using bivariate analyses and also examine the distribution of outcome variables. We will then examine intervention effects over the study using a Poisson GLMM to account for correlations between repeated measurements and an indicator for the intervention group (SMI+I and SMI). As mentioned earlier, we will examine interaction effects as appropriate, including intervention by time.

#### Aim 3: Examine How the Level of Engagement in Intervention Conditions and Characteristics of Engagement Relate to Alcohol Use Outcomes in the 2 Intervention Conditions

##### Hypothesis

Participants in the intervention who have more frequent engagement and more positive valence and arousal will have significantly less alcohol consumption and consequences over 12 months of follow-up than participants with less interaction.

##### Statistical Analysis

We will examine the level of intervention involvement (eg, number of posts) and valence/arousal from the 8-week intervention period as predictors of alcohol outcomes at 3, 6, and 12 months using GLMMs. We will conduct sensitivity analyses by stratifying models by intervention condition. We will create graphs of the outcomes by treatment group to inform how potential variation in the effect of the intervention conditions on the outcome over time is examined in GLMMs (eg, consider interaction terms of condition with time that test for linear or quadratic increases or decreases in effect size over time).

#### Sample Size for Primary Aims

Power was estimated based on an N value of 900 and approximately 75% follow-up rate (conservatively based on our team’s prior alcohol brief intervention, which had >80% compliance with interventions and follow-ups over 12 months) [62], which does not take into account imputations and other strategies for handling missing data without reducing sample size. All power analyses were conducted using G*Power 3.1.7. software and assumed a two-sided test with an alpha of .05. Although we conservatively estimated effect sizes based on the brief intervention literature, we hope that the 8-week intervention period will enhance effect sizes. Although we were not able to locate software for calculating power for GLMMs, we estimated power assuming one follow-up and using traditional statistical tests and anticipate that the greater number of observations will be partially offset by correlation of observations within participant, resulting in similar power. For aim 1, we estimated that we will have >80% power to detect an 11.1% difference between intervention and control groups on alcohol consumption and a 12.5% in alcohol consequences. For aim 2, we estimated we will have >80% power to detect alcohol consumption that is 11.3% lower in the SMI+I group than the SMI group. For aim 3, we estimated we will have >80% power to detect intervention engagement as a continuous variable predicting a reduction in alcohol consumption and consequences.

## Results

Recruitment for the RCT began on January 5, 2017, and was completed on April 20, 2019, with 10 waves of recruitment to enroll the final sample. Across all waves, 11,914 individuals self-administered the web-based screening survey, and we sent baseline invitations to 1541 participants who screened positive on the AUDIT-C and passed initial verification processes. There were 1015 participants who completed the baseline survey; however, 46 individuals did not send a selfie for verification, 8 did not pass selfie verification procedures, 4 indicated they were too busy to join the study, and 2 timed out (did not complete all baseline procedures by group start). Thus, a total of 955 completed all baseline procedures (survey and selfie verification) and were randomized to one of 3 conditions: SMI+I (N=321), SMI (N=321), and attention control (N=313). The 8-week groups were completed in June 2019, and follow-up assessments are ongoing.

There were a total of 5 User Safety Agreement violations during the course of the groups, which resulted in removing a post and reminding participants of this agreement (eg, a public post to the group and/or private message). These violations included 1 individual posting an advertisement for a business, 1 individual posting a personal fundraising page, 1 individual posting a disturbing image, 1 individual using derogatory language regarding mental health, and 2 individuals arguing about politics that included swearing and name calling.

## Discussion

Although social media has been used to deliver interventions addressing other health behaviors [80-82,86], this RCT is one of the first to examine the efficacy of SMIs to reduce risky drinking among adolescents and emerging adults. Given the popularity and daily use of social media among young people [55,56], our intervention capitalizes on a highly used medium that is already routinely a part of their daily lives, unlike prior computerized interventions or alcohol-specific smartphone apps. Furthermore, addressing limitations of prior expensive computer applications that use software that quickly becomes out of date, these SMIs allow for ease of integration into common Web applications by nontechnical staff to facilitate sustainability.

The study protocol described here creates a recipe for future SMIs, as applied to early interventions for substance use. Similar to studies of HIV risk reduction [88], our interventions harness the Facebook feature of secret groups that preserve privacy and facilitate group interaction with other participants in real time, catalyzed by e-coaches who post dynamic content daily. The incentive condition harnesses participants to engage with other participants, which will provide interesting comparison with the nonincentivized intervention condition. In addition, unlike many prior alcohol interventions, which ignore concomitant other drug use, our intervention primarily addresses alcohol while also addressing the use of other drugs and associated health consequences (eg, injury, impaired driving, overdose prevention). Future papers will examine the efficacy of these innovative SMIs, which could have a significant public health impact by altering the alcohol use trajectories of adolescents and emerging adults.

## Abbreviations

AUDIT-C: Alcohol Use Disorders Identification Test-Consumption
BYAACQ: Brief Young Adult Alcohol Consequences Questionnaire
CBT: cognitive behavioral therapy
e-coach: electronic coach
GLMM: generalized linear mixed model
IRB: institutional review board
MI: Motivational Interviewing
MINT: MI Network of Trainers
RCT: randomized controlled trial
SDT: self-determination theory
SMI: social media intervention
SMI+I: social media intervention with incentives
TLFB: Timeline Follow-Back
